# VMAT for Head and Neck Cancer: What Is the Impact of the Optimization Parameters on the Treatment Plan Quality?

**DOI:** 10.3390/medsci14010150

**Published:** 2026-03-19

**Authors:** Evgenia Konstantinou, Efrossyni Lyraraki, Michalis Mazonakis

**Affiliations:** 1 Department of Medical Physics, Faculty of Medicine, University of Crete, 71003 Heraklion, Crete, Greece; 2 Department of Radiotherapy and Oncology, University Hospital of Heraklion, 71110 Heraklion, Crete, Greece

**Keywords:** head and neck cancer, optimization parameters, VMAT-SIB, comparison analysis

## Abstract

Objectives: To evaluate the effectiveness of different optimization parameters on radiotherapy plan quality for seventeen head and neck cancer patients. Methods: Volumetric Modulated Arc Therapy with Simultaneous Integrated Boost (VMAT-SIB) plans, involving up to three tumors, were generated. For each participant, a reference plan (Plan_Ref) was created using dual-arc with 180 control points, 20° gantry-angle increment and 1 cm minimum segment width. Modified plans were developed with dose constraints and optimization settings constant by changing to single-arc, 150 and 200 control points, 0.5 cm minimum segment width, and 30° and 40° gantry-angle increments. These plans were referred to as Plan_Arc1, Plan_CP150, Plan_CP200, Plan_SW0.5, Plan_Inc30, and Plan_Inc40, respectively. D_95%_ of planning target volumes (PTVs), homogeneity index (HI), monitor units (MUs), maximum dose (D_max_) of spinal cord, mandible, and brainstem were recorded. Statistical and Bland–Altman analysis was performed comparing the modified plans to Plan_Ref. Results: Average D_95%_ values for PTV1, PTV2, and PTV3 ranged from 93.13 to 98.82%. Plan_SW0.5 provided superior target coverage and homogeneity with higher MUs than Plan_Ref. Plan_Arc1 significantly reduced PTV coverage and dose homogeneity, while increasing MUs compared with Plan_Ref (*p* < 0.05). The average D_max_ as derived from all planning approaches was up to 43.86 Gy, 65.86 Gy, and 48.85 Gy for spinal cord, mandible and brainstem, respectively. For spinal cord, Plan_Ref led to significantly lower doses compared to Plan_Arc1 and Plan_Inc30, while the brainstem recorded statistically higher D_max_ doses than Plan_Arc1. Significantly higher D_max_ was observed for the mandible using Plan_SW0.5 (*p* < 0.05). However, for D_max_, the comparison plans showed good agreement with Plan_Ref based on Bland–Altman analysis. Conclusions: The VMAT plan quality is strongly affected by the minimum segment width whereas no differences were observed with the modification of the number of control points.

## 1. Introduction

Head and neck cancer remains a global health concern, with approximately 950,000 incidents and 480,000 deaths reported worldwide in 2022 [1]. The locoregional management of these malignancies frequently involves radiation therapy, either as a single modality or in combination with other anticancer modalities [2,3,4,5]. In recent years, advanced radiotherapy techniques have been developed, ensuring precise tumor targeting, while sparing the surrounding organs from radiation exposure [6,7,8].

Radiotherapy, even though targeted therapy, is associated with a substantial increase in toxicities [9,10]. The incidences of radiation-induced toxicities are associated with the radiation exposure of surrounding organs. Minimizing radiation dose to critical organs is crucial to reduce these toxicities. Volumetric Modulated Arc Therapy with Simultaneous Integrated Boost (VMAT-SIB) is commonly employed for head and neck cancer patients. This technique improves treatment plan efficiency along with organ dose sparing and delivery time [11,12,13]. Despite these effective outcomes, further enhancements can be attained through the appropriate selection of optimization parameters such as the number of arcs, control points, minimum segment width and gantry angle increment. The optimization process is essential to minimize the risk of toxicity occurrence while improving plan quality. Specifically, the control points determine the number of generated segments, while the segment width defines the minimum distance between opponent multi-leaf collimator (MLC) leaves, which are adjusted to create segments of varying shapes and sizes. Modifying these parameters affects the treatment complexity, as well as dose conformity and accuracy. The gantry angle increment value divides the total arc length into equal sectors, within which the MLC leaves move in a single direction, reversing in the subsequent sector. Variations in the selected gantry angle increment can influence plan quality, particularly in terms of target dose coverage and homogeneity.

Previous comparative analyses have investigated how different optimization parameters affect treatment quality, indicating their critical role in VMAT planning [14,15,16,17]. Nithya et al. [18] evaluated the effect of increment of gantry and the number of arcs in VMAT planning among esophageal cancer patients. Their investigation focused on a specific head and neck cancer malignancy, evaluating the influence of only two optimization parameters. Another assessment of the optimal selection of minimum segment width in relation to VMAT plan quality and deliverability was contacted by Yoosuf et al. [19]. This work was performed for five different cancers, including brain, head and neck, thorax, pelvis and extremity. Although the importance of these findings is significant, the published literature is limited in the analysis of up to two optimization parameters, either within a single cancer or multiple cancers. To the best of our knowledge, no prior study has systematically evaluated the combined impact of multiple optimization parameters specifically in head-and-neck malignancies within a uniform planning platform (Monaco 6.2.2.0), including agreement analysis via Bland–Altman plots.

The present study aims to investigate the impact of optimization parameters on treatment plan quality for head and neck cancer patients by performing systematic VMAT plan comparisons. This comparative analysis addresses gaps in the existing literature, providing evidence for treatment planning optimization management.

## 2. Materials and Methods

### 2.1. Patients

A total of seventeen head and neck cancer patients with a mean age of 65 ± 10 years were studied. All participants had previously undergone radiotherapy treatment with the VMAT-SIB technique at our institute. Prior to the commencement of this work, all radiation therapy treatments had been completed. Ethical approval for the implementation of this study was granted by the institution’s Ethics Committee of the University Hospital of Heraklion (Approval Code: 2415, Approval Date: 22 January 2024). The patients were diagnosed with malignant neoplasm of various head and neck sites, including six nasopharyngeal, five laryngeal, three lingual, one cutaneous squamous cell carcinoma, one submandibular gland, and one tonsillar case.

### 2.2. Planning Computed Tomography

Planning computed tomography (CT) scans were performed using the Revolution GSI scanner (General Electric Medical Systems, Waukesha, WI, USA). CT images with slice thickness of 2.5 mm were acquired at 120 kV. During the simulation procedure, patients were immobilized in the supine position using individual thermoplastic masks. An experienced radiation oncologist, specialized in the treatment of head and neck cancer, manually delineated the tumor, surrounding lymph node areas and organs at risk (OARs), comprising the spinal cord, mandible, brainstem, parotid glands and right and left cochlea [20,21]. Based on tumor contour, planning target volume (PTV) was defined along with up to two boost target volumes. All the contours were delineated through the use of the treatment planning system (Monaco 6.2.2.0, Elekta AB, Stockholm, Sweden).

### 2.3. Dose Prescription and Dose Constraints

In twelve cases, treatment plans prescribed 70 Gy in 35 fractions of 2.0 Gy to the primary PTV (PTV1), with simultaneous delivery of 63 Gy and 56 Gy in 35 fractions to the secondary (PTV2) and tertiary (PTV3) volumes, respectively. In two other cases, the dose prescription was 70 Gy, 63 Gy, and 59.5 Gy for 35 fractions to PTV1, PTV2, and PTV3, respectively. Two additional cases involved two PTVs and delivered simultaneous doses of 70 Gy and 63 Gy, while one case followed a prescription scheme of 70 Gy and 59.5 Gy. Ninety-five percent of each PTV (D_95%_) was required to deliver at least 95% of the total prescribed dose. The maximum allowable dose (D_max_) was 50 Gy for the spinal cord, 70 Gy for the mandible, and 54 Gy for the brainstem. For the cochlea and the parotids, the mean dose (D_mean_) and the volume receiving 40 Gy (V_40 Gy_) were evaluated due to their overlap with the PTV. The overall volume of parotid glands excluding the organ portions encompassed by the PTVs (Total parotids—PTV) was calculated in accordance with previous works dealing with the sparing of these tissues [22,23].

### 2.4. Treatment Planning

Radiotherapy treatment plans were generated with the aid of the Monaco system for the Infinity linear accelerator (Elekta AB, Stockholm, Sweden), using 6 MV photon beams. The above medical linac is equipped with Agility MLC head featuring 160 tungsten alloy leaves. The projected leaf width at the isocenter is 5 mm. The Monte Carlo algorithm was used for dose calculations, followed by optimization with the fluence optimizer algorithm with a 1% statistical uncertainty per calculation and a 0.3 cm grid spacing. For all plans, the collimator was in 0°. A VMAT-SIB technique was applied in all cases, consisting of a dual arc of 360° rotated in clockwise and counterclockwise directions. The maximum number of control points per arc was set to 180, with a gantry angle increment of 20°, and a minimum segment width of 1 cm. This approach is currently implemented in clinical practice at our department for the treatment of head and neck cancer patients.

To determine the optimal optimization scheme for treatment planning, four different optimization parameters were individually assessed. The examined planning parameters included the number of arcs, control points (150 and 200), minimum segment width (0.5 cm), and gantry angle increment (30° and 40°). For each patient, a total of seven plans were generated, each involving the modification of a single optimization parameter. The importance of the above parameters on VMAT plans for head and neck malignancies has been mentioned in previous works [14,15,16,24,25]. All re-optimized plans were derived from the initial plan, hereafter referred to as Plan_Ref, maintaining consistent dose constraints and optimization settings, except for the parameter under evaluation. Each plan was named according to the modified optimization parameter as Plan_Arc1, Plan_CP150, Plan_CP200, Plan_SW0.5, Plan_Inc30, Plan_Inc40. To ensure accurate comparison across the treatment plans, a normalization to a mean dose of 70 Gy to PTV1 was performed. A summary of all treatment plans and the corresponding parameter values are provided in Table 1. 

### 2.5. Plan Evaluation and Analysis

A dose-volume histogram (DVH) was utilized to evaluate the target coverage and OARs doses. The extracted dosimetric parameters comprised D_95%_ of PTVs, D_max_ of spinal cord, mandible, and brainstem, D_mean_ of of right and left cochlea, and V_40 Gy_ of parotids and monitor units (MUs). For PTV1, three quantitative parameters were calculated for each treatment plan. The homogeneity index (HI) was estimated as the below ratio:

$$HI=\frac{D_{5\%}}{D_{95\%}}$$

where D_5%_ and D_95%_ are the radiation dose to the 5% and 95% of the PTV, respectively. The conformity index (CI) was defined as follows:

$$CI=\frac{{PTV}_{PI}}{V_{PI}}$$

where PTV_PI_ is the target volume covered by the prescription isodose (PI), while V_PI_ refers to the total volume enclosed by the prescription isodose on the CT images. The optimal value of HI and CI is ‘1’.

To evaluate the dose differences in OARs dosimetric parameters between the treatment plans, the percent difference was calculated for each patient using the following formula:

$$D_{ref,i\%}=\frac{{DP}_{i}-{DP}_{ref}}{{DP}_{ref}}\times 100\%$$

where DP_i_ is the dosimetric parameter values of treatment plans (Plan_Arc1; Plan_CP150; Plan_CP200; Plan_SW0.5; Plan_Inc30; Plan_Inc40) and DP_ref_ is the dosimetric parameter values derived from the reference plan (Plan_Ref).

### 2.6. Statistical Analysis

Statistical analysis was performed using the MedCalc software package (version 7.4.4, MedCalc software, Ostend, Belgium). Descriptive statistics, including the mean and standard deviation of the recorded dosimetric parameters, were calculated over all plans. For each OAR dosimetric parameter, the percent difference was also calculated using the formula presented in Section 2.5, and the mean value of the corresponding doses were reported. Utilizing the Shapiro–Wilk test, the normalization analysis was assessed. To compare the reference plan with the remaining treatment plans across the examined dosimetric parameters, paired-sample *t*-test and Wilcoxon signed ranks test were conducted. Statistical significance was defined at the 5% level (*p* < 0.05). For the D_max_ of the spinal cord, mandible and brainstem, the agreement between the dose values from the Plan_Ref and those from the modified plans was calculated through the Bland–Altman statistical method [26]. For each dosimetric parameter, the mean difference (MD) between the values of this parameter as derived from the two VMAT plans was computed. The 95% limits of agreement (LoA) were defined as MD ± 1.96 times the standard deviation of the differences. These values were visualized in the corresponding plots. Each point represents the difference between two paired measurements plotting against their mean value. Good agreement is indicated by points that cluster around the MD line, while points outside the LoA represent outliers.

## 3. Results

### 3.1. PTV Dose Parameters

**Figure 1:**
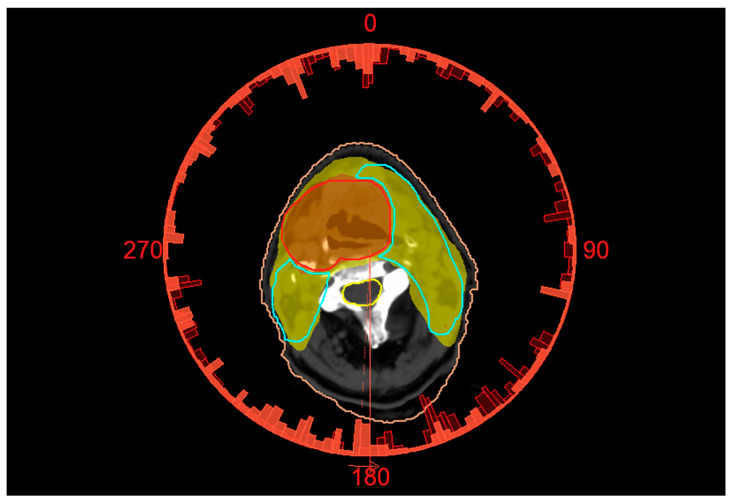
Computed tomography image with contoured structures: PTV1 (red); PTV2 (green) and spinal cord (yellow). Two full arcs of VMAT treatment plan with dose distribution around the two PTVs. The presented isodose areas of orange and yellow correspond to 95% of the prescribed doses of PTV1 and PTV2, respectively.

The reference VMAT plan for a representative patient with lingual cancer, illustrating the isodose distributions covering the target volumes, is shown in Figure 1. Table 2 summarizes the mean values of PTV dosimetric parameters across all the treatment plans. Using the seven irradiation approaches, dose coverage for PTV1, PTV2, and PTV3 ranged from 96.03 to 97.91%, 93.13 to 97.28%, and 96.94 to 98.82%, respectively. The highest D_95%_ values of the three PTVs were observed for Plan_SW0.5, while the lowest target coverage was found for Plan_Arc1. All average HI values remained below 1.04, whereas the average CI values exceeded 0.93. The MU range varied from 615.5 to 986.6, with Plan_SW0.5 exhibiting the highest values among all the treatment plans.

All statistical comparison results are presented in Table 3. For PTV1, the D_95%_ parameter of the Plan_Ref differed significantly from that obtained by Plan_Arc1, Plan_SW0.5, Plan_Inc30, and Plan_Inc40 (*p* < 0.005). Similar differences between Plan_Arc1 and Plan_SW0.5 with Plan_Ref were found for PTV2 (*p* < 0.05). For PTV3, significant differences were detected between Plan_Ref and Plan_Arc1 (*p* = 0.0032). Statistical differences were observed for HI across all the comparisons, except for Plan_CP200 and Plan_Ref. The CI calculated either by the plans with a single arc or by those with a 0.5 cm minimum segment width were significantly lower than the CI derived from the Plan_Ref (*p* < 0.05). Regarding the MUs, all comparisons showed statistically significant differences, except for those between the plans using 150 and 200 control points.

### 3.2. OARs Dose Parameters

The mean values of the dosimetric parameters of the evaluated OARs derived from all the comparisons are listed in Table 2. Among all treatment plans, the range of average D_max_ values were 43.48–43.86 Gy for the spinal cord, 64.83–65.86 Gy for the mandible and 47.74–48.85 Gy for the brainstem. The D_mean_ of right and left cochlea was between 35.45 Gy and 37.24 Gy. Average V_40 Gy_ values for parotids did not exceed 40.61%, with the highest values recorded from Plan_Ref. According to Table 3, significant differences were found in the spinal cord when evaluating Plan_Arc1 and Plan_Inc30 compared to Plan_Ref. For the mandible, D_max_ was significantly higher in Plan_SW0.5 compared to Plan_Ref (*p* = 0.0447). A significant difference was also observed between Plan_Ref and Plan_Arc1 for the brainstem (*p* = 0.0481), with Plan_Arc1 being more favorable in this aspect. Additionally, differences were noted for the parotids across Plan_SW0.5, Plan_Inc30, and Plan_Inc40 compared to the reference plan. In contrast, the cochlea recorded no statistical differences across the six treatment plan comparisons.

Table 4 presents the mean percent differences in OAR dosimetric parameters for the reference plan relative to the six examined plans, ranging from 0.02 to 0.89%, 0.00 to 1.62%, and −2.19 to 0.25% for the spinal cord, mandible, and brainstem, respectively. The average percent differences in the V_40 Gy_ of the parotid ranged from −6.42 to –0.39%, while the D_mean_ for the right and left cochlea were between −3.48 and 1.31%.

**Figure 2:**
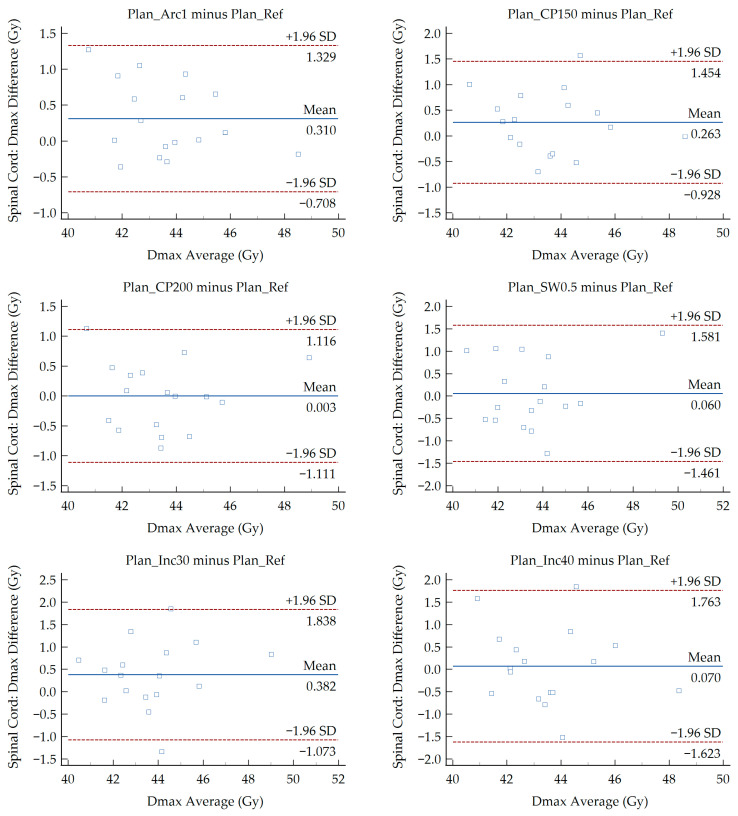
Bland–Altman plots showing the difference in the D_max_ of the spinal cord between the Plan_Ref and the six examined VMAT plans.

**Figure 3:**
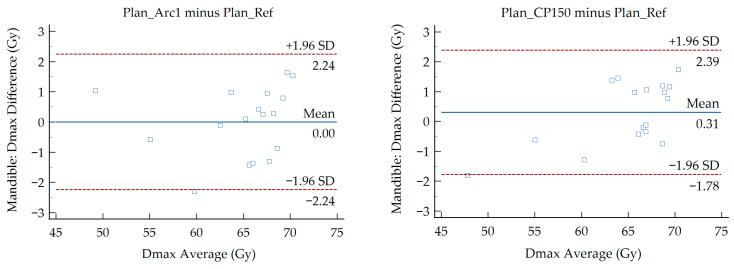

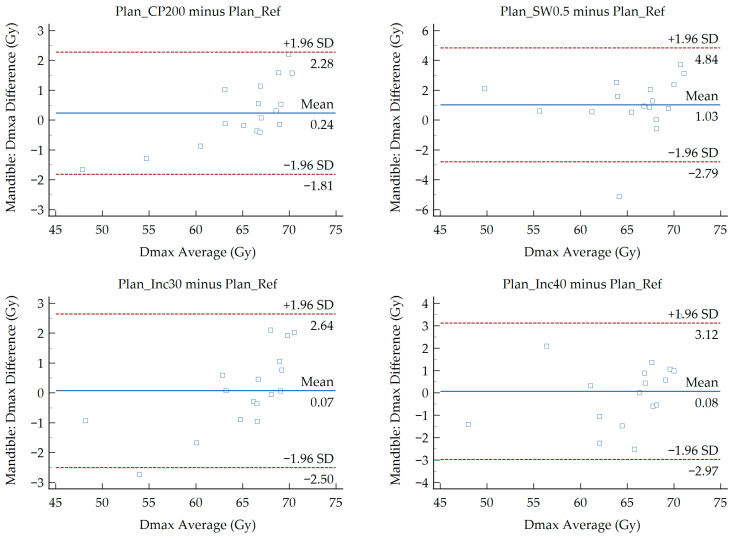
Bland–Altman plots showing the difference in the D_max_ of the mandible between the Plan_Ref and the six examined VMAT plans.

**Figure 4:**
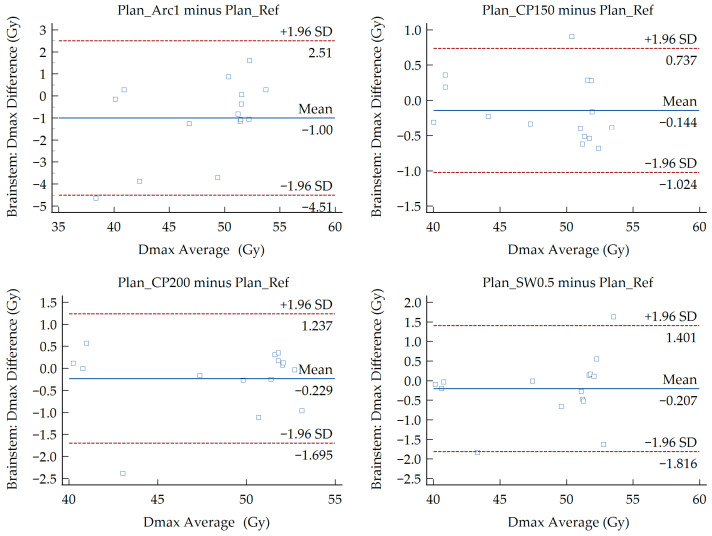

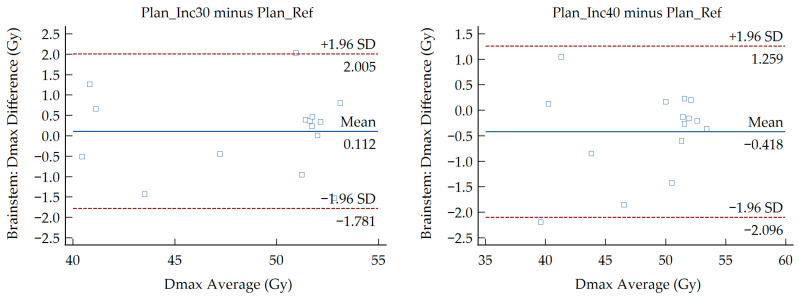
Bland–Altman plots showing the difference in the D_max_ of the brainstem between the Plan_Ref and the six examined VMAT plans.

The results of Bland–Altman analysis are presented in Figure 2, Figure 3 and Figure 4. These plots evaluate the agreement between the six modified VMAT plans and the reference plan for the Dmax of the spinal cord, mandible, and brainstem. Each point represents the average Dmax of the compared plans plotted against the difference of Dmax between them. Values for the MD and LoA are provided on each figure. The OARs exhibited a D_max_ MD ranging from −1.00 to 1.03 Gy and LoA between −4.51 and 4.84 Gy. The LoA defines the range where 95% of differences lie. No more than one data point falls outside the LoA in the majority of the generated Bland–Altman plots. In two plots, one point lies outside the LoA while another lies exactly on the LoA. These correspond to the spinal cord (Plan_Inc30 minus Plan_Ref) and the brainstem (Plan_SW0.5 minus Plan_Ref).

## 4. Discussion

The design of radiotherapy treatment plan aims to ensure therapeutic effectiveness while minimizing radiation-induced toxicity. Despite the technological improvements provided by advanced techniques, continuous optimization of treatment plans remains essential. The selection of optimization parameters influences the treatment plan quality and should be evaluated in terms of target coverage and the radiation dose delivered to surrounding organs.

This work presents a comparative evaluation of VMAT plans by investigating the impact of four optimization parameters on target coverage and radiation exposure to critical surrounding organs. The analyzed parameters included the number of arcs, control points (150 and 200), minimum segment width (0.5 cm), and gantry angle increment (30° and 40°). The study cohort comprised seventeen patients with various head and neck malignancies who had completed radiation therapy. For each patient, a reference plan was generated using the VMAT-SIB technique, involving up to three targets. Six modified plans, each implementing a single optimization parameter change, were made and subsequently compared to the reference. All plans were created for the purpose of this study, and no treatment deliverability tests were performed.

All treatment plans attained over 95% target coverage. The only exception was observed for PTV2 when a single arc VMAT plan was generated. The use of Plan_SW0.5 provided significantly higher target dose coverage for both PTV1 and PTV2, with improved homogeneity in PTV1 compared to Plan_Ref. However, the number of MUs was significantly higher for Plan_SW0.5 (986.6 vs. 652.9, *p* < 0.0001). The superiority of the reference plan over the 30° and 40° gantry angle increment plans was demonstrated with respect to PTV1 dose coverage and HI (Table 2 and Table 3). However, Plan_In30 and Plan_Inc40 achieved significantly lower MU values relative to Plan_Ref. Plan_Arc1 resulted in significantly lower target dose coverage and dose conformity in PTV1, with statistically higher MUs and dose homogeneity compared to Plan_Ref. No significant differences were found for the plans utilizing 150 and 200 control points, relative to the reference plan, except for the HI between Plan_CP150 and Plan_Ref. These findings, obtained by either increasing or decreasing the number of control points, clearly reveal the negligible impact of control points on plan quality. This can be attributed to the lower control points utilized during the optimization procedure, compared to the maximum allowable. For example, each Plan_CP200 was generated with a maximum number of 200 control points per arc. This implies a maximum total number of points of 400 in the dual arc. However, a much smaller number of control points was used by the algorithm. The average number of control points used during optimization process was only 236 ± 19. Similar observations were made for Plan_Ref and Plan_CP150. These differences reflect the algorithm optimizer’s ability to adequately modulate dose even with fewer control points.

Individual radiotherapy treatment plans are generated primarily to satisfy the clinical planning objectives. Beyond these aspects, other factors are also considered when evaluating plan quality. Higher target dose coverage can result in increased MUs. Our results demonstrate that using reduced segment width and a single arc increased MUs by up to 51%, despite the improved target coverage. Increased MUs are generally associated with higher leakage radiation and longer treatment delivery time, potentially leading to an increased risk of radiation-induced secondary malignancies [27]. Furthermore, highly modulated VMAT plans may increase delivery complexity, which may affect the deliverability accuracy. However, in our study, plans with larger gantry angle increments showed a reduction in MUs of up to 6% compared with the reference plan, while the D_95%_ coverage for the three PTVs decreased by less than 0.6% in both Plan_Inc30 and Plan_Inc40. Such dose coverage differences, which correspond to an absolute difference of less than 1% of the prescription dose, are considered clinically negligible in head and neck VMAT planning. Although statistically significant, the magnitude of observed differences in MUs and D_95%_ among the evaluated plans remained within clinically acceptable tolerances.

This study also assessed the effect of the aforementioned optimization parameters on dose sparing in regard to OARs. As shown in Table 4, the absolute percentage difference remained below 1% for the majority of the OARs across all comparisons. Higher percentage differences were recorded in the comparison between Plan_Ref and Plan_Arc1 for the brainstem and left cochlea (−2.19% and −3.48%, respectively). Similarly, for the parotid glands the reference plan obtained higher differences compared to those of 0.5 cm minimum segment width and a gantry angle increment of 30° and 40°. According to Table 2 and Table 3, the use of Plan_Arc1 and Plan_Inc30 led to significantly higher D_max_ values for the spinal cord compared with Plan_Ref (*p* < 0.05). A significant difference was also observed for the mandible when a 0.5 cm minimum segment width was applied. For the brainstem, the Plan_Arc1 resulted in significantly greater brainstem dose sparing when compared with Plan_Ref (47.74 Gy vs. 48.74 Gy, *p* = 0.0481). Significantly reduced doses to the parotid glands were achieved utilizing a minimum segment width of 0.5 and gantry angle increments of 30° and 40°.

Regardless of the presence of significant and non-significant differences among the treatment plans, the above findings do not demonstrate the dosimetric agreement between the different VMAT planning approaches. To investigate the potential interchangeability of the modified plans with the reference plan, a Bland–Altman analysis was performed. The analysis revealed minor MD for the D_max_ of the OARs under investigation. Absolute MD values below 0.50 Gy were recorded for all structures, whereas the man-dible and brainstem showed higher MD values of 1.03 Gy and 1.00 Gy in the Plan_SW0.5 and Plan_Arc1 comparisons, respectively. The MD values for the brainstem were negative in all comparisons except for Plan_Inc30 relative to Plan_Ref, whereas the corresponding values for the spinal cord and mandible were positive. A negative MD demonstrates that the reference plan predicted higher brainstem doses than the modified plan, while a potive MD reflects the opposite. In accordance with the presented plots (Figure 2, Figure 3 and Figure 4), the limits were found to be relatively narrow compared with the dose values of each parameter. For the D_max_ of the spinal cord, the widest LoA were derived from the comparison between Plan_Inc40 and Plan_Ref. These limits indicate that for 95% of cases, the D_max_ deviations between the reference and modified VMAT plans lie within the recorded ranges. For instance, in that case the LoA ranged from −1.62 to 1.76 Gy, with a MD of 0.07 Gy. Τhe average D_max_ values for Plan_Ref and Plan_Inc40 were 43.48 and 43.55 Gy, respectively. Considering a ± 2 Gy tolerance threshold, both values remain below the commonly accepted spinal cord tolerance limit (D_max_ < 50 Gy). Therefore, the observed differences can be considered clinically acceptable. This indicates that the modified plans provide organ doses comparable to the reference plan. Plan comparisons, including reduced minimum segment width for the mandible and single-arc delivery for the brainstem, were associated with wider LoA (−2.79 to 4.84 Gy and −4.51 to 2.51 Gy, respectively). These differences can be attributed to patient-specific anatomical variations along with reduced modulation potential of the 0.5 cm segment width and the single-arc delivery, respectively. Nevertheless, the observed deviations remained within clinically accepted dose thresholds (mandible D_max_ < 70 Gy, brainstem D_max_ < 54 Gy), indicating that these modified plans maintain acceptable sparing of OARs.

Several studies have investigated the impact of different optimization parameters on VMAT plan quality. However, to the best of our knowledge, no previous studies have simultaneously examined multiple optimization parameters across different head and neck malignancies within a single analysis. Shen et al. [28] assessed the impact of gantry angle increments of 10°, 20°, 30°, and 40° on VMAT delivery for breast cancer, with lower angle increments (10° and 20°) being preferable in relation to plan quality and delivery efficiency. However, in a similar study by Chen et al. [14], greater angles of 30° and 40° were recommended. Their findings were obtained from VMAT planning for cervical cancer, showing that higher angles yielded significantly reduced MUs, with 30° providing slightly better target coverage against the 20° angle. Our work revealed that the increment of 20° is preferable, based on our results for VMAT planning of head and neck cancer, with respect to target volume coverage and homogeneity as opposed to 30° and 40° increments. The work of Thimothy et al. [15] compared VMAT plans generated with minimum segment widths of 0.5 cm, 1.0 cm, and 1.5 cm for prostate cancer. Significantly lower MUs and improved dose homogeneity, without affecting OARs sparing, were provided by decreasing the widths. Considering the plan quality and treatment efficiency, the 1.0 cm minimum segment width was deemed optimal. Our study resulted in comparable findings. A comparison of single- and dual-arc VMAT plans was conducted by Lee et al. [17]. The dual-arc plans resulted in significantly superior PTV coverage and OAR sparing when compared to single-arc plans, consistent with our results.

There are some limitations to this work. First, our results were derived from treatment planning data and were not independently verified. Potential discrepancies between the planned and delivered doses, and therefore the delivery accuracy, were not investigated, particularly for Plan_SW0.5. Narrow segment widths, such as the 0.5 cm width used, may also increase the sensitivity of dose delivery to patient motion. Quality assurance tests would be required to assess plan deliverability. Second, a larger cohort of patients is needed for external validity of our findings. In addition, those comparisons were conducted exclusively on head and neck cancer patients. Further investigation involving other cancer anatomical sites are required to assess the generalizability of these findings. No correction for multiple comparisons was applied, as the study focused on a limited number of pre-specified comparisons. Another limitation for this work is that all treatment plans were performed with the aid of the Monaco system. Additionally, an assessment of normal tissue complication probabilities (NTCP) or other radiobiological indices would add valuable input to the current dosimetric analysis. It would also be relevant to evaluate plan complexity metrics and treatment delivery time, which offer the advantage of offering insight into the practical implementation of the plans. Our study focused on individual comparisons between the reference plan and each modified plan. The potential of pairwise comparisons among all seven plans could provide a more comprehensive assessment of plan quality.

## 5. Conclusions

The optimum selection of optimization parameters can significantly affect VMAT plan quality, treatment efficiency, and clinical workflow. This comparative study suggests that dual-arc plans may be preferred for achieving optimal target coverage, while a minimum segment width of 1 cm appears to provide a clinically efficient compromise between dose homogeneity and monitor units. Plans with 30° and 40° gantry increment angles significantly reduced the number of MUs compared to the reference plan without clinically meaningful differences in target coverage. No notable differences were observed regarding the plans of 150 and 200 control points. These findings provide practical guidance, based on observed differences and agreement measures. Health care professionals, including radiation oncologists, medical physicists and medical dosimetrists, may consider this guidance for selecting the appropriate optimization parameters in the VMAT planning of head and neck malignancies.

## Figures and Tables

**Table 1:** Radiotherapy treatment plans with the examined optimization parameters.

Plan Name	Arc	Control Points/Arc	Minimum Segment Width (cm)	Increment (°)
Plan_Ref	2	180	1	20
Plan_Arc1	1	180	1	20
Plan_CP150	2	150	1	20
Plan_CP200	2	200	1	20
Plan_SW0.5	2	180	0.5	20
Plan_Inc30	2	180	1	30
Plan_Inc40	2	180	1	40

Plan_Ref: reference plan; Plan_Arc1: plan with a single arc; Plan_CP150: plan with 150 control points; Plan_CP200: plan with 200 control points; Plan_SW0.5: plan with 0.5 cm minimum segment width; Plan_Inc30: plan with 30° gantry angle increment; Plan_Inc40: plan with 40° gantry angle increment.

**Table 2:** Dosimetric parameters for the examined structures as derived from the seventeen patients and for the seven treatment plans. The values are shown as mean ± one standard deviation.

Structure	Parameter	Mean ± Standard Deviation						
		Plan_Ref	Plan_Arc1	Plan_CP150	Plan_CP200	Plan_SW0.5	Plan_Inc30	Plan_Inc40
PTV1	D_95%_ (%)	97.33 ± 2.05	96.03 ± 2.53	96.95 ± 2.28	97.11 ± 2.01	97.91 ± 1.72	96.78 ± 2.28	96.81 ± 2.22
PTV2	D_95%_ (%)	96.57 ± 1.38	93.13 ± 2.28	96.19 ± 1.75	96.32 ± 1.58	97.28 ± 1.81	96.15 ± 1.62	96.35 ± 1.72
PTV3	D_95%_ (%)	98.80 ± 1.01	96.94 ± 2.79	98.48 ± 1.46	98.70 ± 1.25	98.82 ± 1.19	98.56 ± 1.37	98.43 ± 1.50
Spinal cord	D_max_ (Gy)	43.48 ± 1.96	43.79 ± 1.79	43.74 ± 1.90	43.48 ± 1.97	43.54 ± 2.10	43.86 ± 2.07	43.55 ± 1.85
Mandible	D_max_ (Gy)	64.83 ± 5.52	64.83 ± 5.68	65.14 ± 6.19	65.06 ± 6.30	65.86 ± 5.74	64.90 ± 6.41	64.91 ± 6.03
Brainstem	D_max_ (Gy)	48.74 ± 4.81	47.74 ± 5.64	48.60 ± 4.70	48.51 ± 4.86	48.54 ± 5.00	48.85 ± 4.84	48.32 ± 4.97
Parotids	V_40 Gy_ (%)	40.61 ± 9.36	40.04 ± 9.36	40.20 ± 10.09	40.49 ± 9.78	38.03 ± 9.55	39.05 ± 9.84	38.97 ± 9.18
Right Cochlea	D_mean_ (Gy)	35.87 ± 20.33	36.00 ± 20.27	35.57 ± 20.17	35.99 ± 20.26	35.45 ± 20.14	36.01 ± 20.09	36.01 ± 20.01
Left Cochlea	D_mean_ (Gy)	36.81 ± 21.39	36.11 ± 21.41	36.71 ± 20.93	37.15 ± 21.53	37.24 ± 21.71	36.95 ± 21.01	37.00 ± 21.74
HI		1.03 ± 0.02	1.04 ± 0.03	1.03 ± 0.02	1.03 ± 0.02	1.02 ± 0.02	1.03 ± 0.02	1.03 ± 0.02
CI		0.96 ± 0.02	0.94 ± 0.03	0.95 ± 0.03	0.95 ± 0.03	0.93 ± 0.04	0.95 ± 0.02	0.96 ± 0.03
MUs		652.9 ± 61.6	733.9 ± 76.0	638.9 ± 61.8	700.0 ± 62.2	986.6 ± 103.3	618.4 ± 64.3	615.5 ± 62.6

Plan_Ref: reference plan; Plan_Arc1: plan with a single arc; Plan_CP150: plan with 150 control points; Plan_CP200: plan with 200 control points; Plan_SW0.5: plan with 0.5 cm minimum segment width; Plan_Inc30: plan with 30° gantry angle increment; Plan_Inc40: plan with 40° gantry angle increment; D_95%_: target percentage receiving 95% of total dose; D_max_: maximum dose; V_40 Gy_: organ volume receiving more than 40 Gy; D_mean_: mean dose; HI: homogeneity index; CI: conformity index; MUs: monitor units.

**Table 3:** Results of statistical comparison of the dose parameters obtained by the reference plan and the six examined treatment plans.

Structure	Parameter	*p*-Value					
		Plan_Arc1 Minus Plan_Ref	Plan_CP150 Minus Plan_Ref	Plan_CP200 Minus Plan_Ref	Plan_SW0.5 Minus Plan_Ref	Plan_Inc30 Minus Plan_Ref	Plan_Inc40 Minus Plan_Ref
PTV1	D_95%_	*p* = 0.0001	NS ^a^	NS	*p* = 0.0074	*p* = 0.0039	*p* = 0.0031
PTV2	D_95%_	*p* < 0.0001	NS ^a^	NS	*p* = 0.0029	NS	NS ^a^
PTV3	D_95%_	*p* = 0.0032 ^a^	NS ^a^	NS	NS	NS ^a^	NS ^a^
Spinal cord	D_max_	*p* = 0.0256	NS	NS	NS	*p* = 0.0497	NS
Mandible	D_max_	NS	NS	NS	*p* = 0.0447 ^a^	NS	NS
Brainstem	D_max_	*p* = 0.0481	NS	NS ^a^	NS	NS	NS
Parotids	V_40 Gy_ (%)	NS	NS	NS	*p* = 0.0017	*p* = 0.0158	*p* = 0.0328
Right Cochlea	D_mean_ (Gy)	NS ^a^	NS	NS	NS	NS	NS
Left Cochlea	D_mean_ (Gy)	NS	NS ^a^	NS ^a^	NS	NS	NS ^a^
HI		*p* = 0.0002	*p* = 0.0445 ^a^	NS	*p* = 0.0083	*p* = 0.0040	*p* = 0.0023
CI		*p* = 0.0050	NS	NS ^a^	*p* = 0.0002	NS	NS
MUs		*p* < 0.0001	NS	NS	*p* < 0.0001	*p* = 0.0087	*p* = 0.0208

Plan_Ref: reference plan; Plan_Arc1: plan with a single arc; Plan_CP150: plan with 150 control points; Plan_CP200: plan with 200 control points; Plan_SW0.5: plan with 0.5 cm minimum segment width; Plan_Inc30: plan with 30° gantry angle increment; Plan_Inc40: plan with 40° gantry angle increment; D_95%_: target percentage receiving 95% of total dose; D_max_: maximum dose; V_40 Gy_: organ volume receiving more than 40 Gy; D_mean_: mean dose; HI: homogeneity index; MUs: monitor units; NS: non-significant (*p* > 0.05). ^a^: non-parametric test.

**Table 4:** Calculated percent difference in OAR dosimetric parameters between the reference plan and six modified plans.

Structure	Parameter	*Percent Difference (%)*					
		Plan_Arc1 Minus Plan_Ref	Plan_CP150 Minus Plan_Ref	Plan_CP200 Minus Plan_Ref	Plan_SW0.5 Minus Plan_Ref	Plan_Inc30 Minus Plan_Ref	Plan_Inc40 Minus Plan_Ref
Spinal Cord	D_max_	0.74 *	0.62	0.02	0.14	0.89 *	0.19
Mandible	D_max_	0.00	0.38	0.26	1.62 *	0.00	0.08
Brainstem	D_max_	−2.19 *	−0.27	−0.47	−0.45	0.25	−0.88
Parotids	V_40 Gy_	−1.12	−1.17	−0.39	−6.42 *	−4.06 *	−3.83 *
Right Cochlea	D_mean_	0.25	−0.89	1.18	−0.17	0.90	1.31
Left Cochlea	D_mean_	−3.48	0.43	0.23	0.64	0.97	−0.14

Plan_Ref: reference plan; Plan_Arc1: plan with a single arc; Plan_CP150: plan with 150 control points; Plan_CP200: plan with 200 control points; Plan_SW0.5: plan with 0.5 cm minimum segment width; Plan_Inc30: plan with 30° gantry angle increment; Plan_Inc40: plan with 40° gantry angle increment; D_max_: maximum dose; V_40 Gy_: organ volume receiving more than 40 Gy; D_mean_: mean dose. * Statistically significant differences (*p* < 0.05).

## Data Availability

The original contributions presented in this study are included in the article. Further inquiries can be directed to the corresponding author.

## References

[1] Bray F., Laversanne M., Sung H., Ferlay J., Siegel R.L., Soerjomataram I., Jemal A. (2024). Global cancer statistics 2022: GLOBOCAN estimates of incidence and mortality worldwide for 36 cancers in 185 countries. CA Cancer J. Clin..

[2] Anderson G., Ebadi M., Vo K., Novak J., Govindarajan A., Amini A. (2021). An updated review on head and neck cancer treatment with radiation therapy. Cancers.

[3] Zhang E.J., Knox M., Veness M.J., Abdul-Razak M., Wong E., Hwang E.J., Carlino M., Sundaresan P. (2025). Outcomes With Radiation Therapy as Primary Treatment for Unresectable Cutaneous Head and Neck Squamous Cell Carcinoma. Clin. Oncol..

[4] Chow L.Q. (2020). Head and neck cancer. N. Engl. J. Med..

[5] Zhang S., Zeng N., Yang J., He J., Zhu F., Liao W., Xiong M., Li Y. (2023). Advancements of radiotherapy for recurrent head and neck cancer in modern era. Radiat. Oncol..

[6] Mazonakis M., Kachris S., Tolia M., Damilakis J. (2023). NTCP calculations of five different irradiation techniques for the treatment of thymoma. Curr. Oncol..

[7] Konstantinou E., Varveris A., Solomou G., Antoniadis C., Tolia M., Mazonakis M. (2024). Radiation dose to critical cardiac structures from three-dimensional conformal radiation therapy (3D-CRT), Intensity-Modulated Radiation Therapy (IMRT) and Volumetric Modulated Arc Therapy (VMAT) techniques for left-sided breast cancer. J. Pers. Med..

[8] Webster M., Podgorsak A., Li F., Zhou Y., Jung H., Yoon J., Dona Lemus O., Zheng D. (2025). New approaches in radiotherapy. Cancers.

[9] Mathew J.M., Ringash J., Su J., Levin W., Bratman S., Cho B.J., Hahn E., Abdalaty A.H., Hope A., Kim J. (2025). Risk factors and survival impact of severe radiation-related late toxicities in head and neck cancer–a cohort study. Lancet Reg. Health Am..

[10] Brook I. (2020). Late side effects of radiation treatment for head and neck cancer. Radiat. Oncol. J..

[11] Osborn J. (2017). Is VMAT beneficial for patients undergoing radiotherapy to the head and neck?. Radiography.

[12] Chen D., Cai S.B., Soon Y.Y., Cheo T., Vellayappan B., Tan C.W., Ho F. (2023). Dosimetric comparison between Intensity Modulated Radiation Therapy (IMRT) vs dual arc Volumetric Arc Therapy (VMAT) for nasopharyngeal cancer (NPC): Systematic review and meta-analysis. J. Med. Imaging Radiat. Sci..

[13] Cilla S., Deodato F., Digesù C., Macchia G., Picardi V., Ferro M., Sallustio G., De Spirito M., Piermattei A., Morganti A.G. (2014). Assessing the feasibility of volumetric-modulated arc therapy using simultaneous integrated boost (SIB-VMAT): An analysis for complex head-neck, high-risk prostate and rectal cancer cases. Med. Dosim..

[14] Chen A., Li Z., Chen L., Lin M., Li B., Chen F. (2019). The influence of increment of gantry on VMAT plan quality for cervical cancer. J. Radiat. Res. Appl. Sci..

[15] Thimothy G., Joan M., Vijay A. (2025). Enhancing Treatment Efficiency: Investigating the Optimal Segment Width in Volumetric Modulated Arc Therapy for Prostate Cancer Management. J. Med. Phys..

[16] Kumar D., Pradhan A., Singh L.M. (2021). A comparative study of the dosimetric impact on IMRT planning with VMAT plans using a varying number of arcs in prostate cancer. J. Phys. Conf. Ser..

[17] Lee T.F., Ting H.M., Chao P.J., Fang F.M. (2012). Dual arc volumetric-modulated arc radiotherapy (VMAT) of nasopharyngeal carcinomas: A simultaneous integrated boost treatment plan comparison with intensity-modulated radiotherapies and single arc VMAT. Clin. Oncol..

[18] Nithya L., Raj N.A.N., Rathinamuthu S., Sharma K., Pandey M.B. (2014). Influence of increment of gantry angle and number of arcs on esophageal volumetric modulated arc therapy planning in Monaco planning system: A planning study. J. Med. Phys..

[19] Yoosuf A.M., Ahmad M.B., AlShehri S., Alhadab A., Alqathami M. (2021). Investigation of optimum minimum segment width on VMAT plan quality and deliverability: A comprehensive dosimetric and clinical evaluation using DVH analysis. J. Appl. Clin. Med. Phys..

[20] Grégoire V., Evans M., Le Q.T., Bourhis J., Budach V., Chen A., Eisbruch A., Feng M., Giralt J., Gupta T. (2018). Delineation of the primary tumour clinical target volumes (ctv-p) in laryngeal, hypopharyngeal, oropharyngeal and oral cavity squamous cell carcinoma: Airo, caca, dahanca, eortc, georcc, gortec, hknpcsg, hncig, iag-kht, lprhht, ncic ctg, ncri, nrg oncology, phns, sbrt, somera, sro, sshno, trog consensus guidelines. Radiother. Oncol..

[21] Brouwer C.L., Steenbakkers R.J., Bourhis J., Budach W., Grau C., Grégoire V., Van Herk M., Lee A., Maingon P., Nutting C. (2015). CT-based delineation of organs at risk in the head and neck region: DAHANCA, EORTC, GORTEC, HKNPCSG, NCIC CTG, NCRI, NRG Oncology and TROG consensus guidelines. Radiother. Oncol..

[22] Bandlamudi B.P., Sharan K., Yathiraj P.H., Singh A., Reddy A., Fernandes D.J., Srinivasa V.M. (2018). A study on the impact of patient-related parameters in the ability to spare parotid glands by intensity-modulated radiotherapy for head and neck squamous cell carcinomas. J. Cancer Res. Ther..

[23] Zhang H., Cao Y., Antone J., Riegel A.C., Ghaly M., Potters L., Jamshidi A. (2019). A model-based method for assessment of salivary gland and planning target volume dosimetry in volumetric-modulated arc therapy planning on head-and-neck cancer. J. Med. Phys..

[24] Wang Y., Chen L., Zhu F., Guo W., Zhang D., Sun W. (2018). A study of minimum segment width parameter on VMAT plan quality, delivery accuracy, and efficiency for cervical cancer using Monaco TPS. J. Appl. Clin. Med. Phys..

[25] Tol J.P., Dahele M., Slotman B.J., Verbakel W.F. (2015). Increasing the number of arcs improves head and neck volumetric modulated arc therapy plans. Acta Oncol..

[26] Bland J.M., Altman D.G. (2010). Statistical methods for assessing agreement between two methods of clinical measurement. Int. J. Nurs. Stud..

[27] Hall E.J., Wuu C.S. (2003). Radiation-induced second cancers: The impact of 3D-CRT and IMRT. Int. J. Radiat. Oncol. Biol. Phys..

[28] Shen C., Liao H., Wang Q., Liu H., Sun X. (2024). Effects of Inc parameter on quality of VMAT radiotherapy plans for right breast cancer based on dosimetric study. Medicine.

